# Biodegradation of 2-chloro-4-nitrophenol via a hydroxyquinol pathway by a Gram-negative bacterium, *Cupriavidus* sp. strain CNP-8

**DOI:** 10.1186/s13568-018-0574-7

**Published:** 2018-03-20

**Authors:** Jun Min, Jinpei Wang, Weiwei Chen, Xiaoke Hu

**Affiliations:** 1 Key Laboratory of Coastal Biology and Bioresource Utilization, Yantai Institute of Coastal Zone Research, Chinese Academy of Sciences, Yantai, 264003 China; 2 Key Laboratory of Agricultural and Environmental Microbiology, Wuhan Institute of Virology, Chinese Academy of Sciences, Wuhan, 430071 China

**Keywords:** 2-Chloro-4-nitrophenol, Hydroxyquinol pathway, Kinetics, Chemotaxis, *Cupriavidus* sp. strain CNP-8

## Abstract

*Cupriavidus* sp. strain CNP-8 isolated from a pesticide-contaminated soil was able to utilize 2-chloro-4-nitrophenol (2C4NP) as a sole source of carbon, nitrogen and energy, together with the release of nitrite and chloride ions. It could degrade 2C4NP at temperatures from 20 to 40 °C and at pH values from 5 to 10, and degrade 2C4NP as high as 1.6 mM. Kinetics assay showed that biodegradation of 2C4NP followed Haldane substrate inhibition model, with the maximum specific growth rate (*μ*_max_) of 0.148/h, half saturation constant (*K*_s_) of 0.022 mM and substrate inhibition constant (*K*_i_) of 0.72 mM. Strain CNP-8 was proposed to degrade 2C4NP with hydroxyquinol (1,2,4-benzenetriol, BT) as the ring-cleavage substrate. The 2C4NP catabolic pathway in strain CNP-8 is significant from those reported in other Gram-negative 2C4NP utilizers. Enzymatic assay indicated that the monooxygenase initiating 2C4NP catabolism had different substrates specificity compared with previously reported 2C4NP monooxygenations. Capillary assays showed that strain CNP-8 exhibited metabolism-dependent chemotactic response toward 2C4NP at the optimum concentration of 0.5 mM with a maximum chemotaxis index of 37.5. Furthermore, microcosm studies demonstrated that strain CNP-8, especially the pre-induced cells, could remove 2C4NP rapidly from the 2C4NP-contaminated soil. Considering its adaptability to pH and temperature fluctuations and great degradation efficiency against 2C4NP, strain CNP-8 could be a promising candidate for the bioremediation of 2C4NP-contaminated sites.

**Electronic supplementary material:**

The online version of this article (10.1186/s13568-018-0574-7) contains supplementary material, which is available to authorized users.

## Introduction

Chloronitrophenols (CNPs) are typical representatives of chlorinated nitroaromatics, which are widely utilized in synthesizing pesticides, fungicides, drugs, dyes, among others (Arora et al. 2012a, b). The natural formation of CNPs is extremely rare, and most of these xenobiotics in the environment are mainly coming from their manufacture and use, and the chemical or biological degradation of their derivatives. As the most common isomer of CNPs, 2-chloro-4-nitrophenol (2C4NP) is used for production of herbicide dicapthon and fungicide nitrofungin (Min et al. 2014). Due to its water solubility and high mobility, 2C4NP has caused serious contamination to agricultural soils and water resources, and has caused severe health effects to humans and animals (Arora et al. 2014a). Therefore, removal of 2C4NP from the environment has recently aroused wide concern.

In the past decade, several physico-chemical methods have been reported on the degradation of 2C4NP (Priya and Madras 2006; Verma et al. 2015); however, these methods are cost-consuming and do not yield complete mineralization of 2C4NP. Bioremediation process, which is more environmental-friendly and cost-effective in comparison with physico-chemical methods, has turned out to be a promising strategy for degradation of various nitrophenol pollutants (Chi et al. 2013; Wang et al. 2014; Min et al. 2017a; Xu and Zhou 2017). Moreover, microbial-based processes can be also effectively integrated with the activated sludge system which was already used in the conventional effluent treatment plants.

Although 2C4NP is released into the environment for a relatively short period, several microorganisms have evolved their ability to degrade this xenobiotic (Ghosh et al. 2010; Arora and Jain 2011; Pandey et al. 2011; Arora and Jain 2012; Tiwari et al. 2017). The Gram-negative *Burkholderia* sp. strain SJ98 degraded 2C4NP with chlorohydroquinone (CHQ) as the ring-cleavage compound (Min et al. 2014), whereas the Gram-positive *Rhodococcus imtechensis* RKJ 300 degraded 2C4NP with hydroxyquinol (1,2,4-benzenetriol, BT) as the ring-cleavage compound (Min et al. 2016). *Burkholderia* sp. RKJ800, another Gram-negative 2C4NP-utilizer, was reported to initiate 2C4NP degradation with formation of CHQ, which was then dechlorinated to hydroquinone (HQ) (Arora and Jain 2012). Due to its high toxicity, 2C4NP with concentration more than 0.5 mM was reported to inhibit the growth of previously reported 2C4NP-utilizers (Arora and Jain 2012; Tiwari et al. 2017). Furthermore, most 2C4NP-utilizers were reported to degrade 2C4NP at neutral pH. Industrial wastewaters, especially the pesticide production wastewaters, may contain high concentration of 2C4NP and the pH often varies widely. Thus, the search for new microorganisms that can adapt to a wide pH range and degrade 2C4NP at high concentration is still of great scientific and industrial significance.

In this study, *Cupriavidus* sp. strain CNP-8 has ability to adapt a wide range of pH and temperature and utilize 2C4NP up to a concentration of 1.6 mM, which is apparently higher than the maximum concentration degraded by previous 2C4NP-utilizers. Strain CNP-8 was proposed to degrade 2C4NP via the BT pathway, which was reported in a Gram-negative 2C4NP-degrading bacterium for the first time. The kinetics of 2C4NP degradation and the chemotaxis of 2C4NP by this strain was also investigated. Microcosm studies demonstrated that strain CNP-8 could be an efficient candidate for bioremediation application.

## Materials and methods

### Bacterial strain, media and chemicals

Strain CNP-8 was recently isolated from the pesticide-contaminated soil collected from Yantai, Shandong, China (Min et al. 2017b). It has been deposited in the China Center for Type Culture Collection (Wuhan) with accession number CCTCC M 2017546. Compounds 2C4NP, 2-chloro-5-nitrophenol (2C5NP), 4-chloro-2-nitrophenol (4C2NP), 5-chloro-2-nitrophenol (5C2NP), BT, CHQ, *para*-nitrophenol (PNP) and *meta*-nitrophenol (MNP) were purchased from Sigma Chemical Co.

### Biodegradation experiments

Biodegradation experiments were carried out in 250 mL Erlenmeyer flasks containing 100 mL MSM with 2C4NP as a sole source of carbon, nitrogen and energy. The 2C4NP degradation ability of strain CNP-8 was determined by monitoring the bacterial growth (OD_600_) and 2C4NP consumption. To investigate the effect of pH and temperature on 2C4NP degradation, biodegradation experiments were performed at different pH (4–10) and temperatures (20–40 °C). Glucose (0.2, 0.5 or 5 g/L) was added into the MSM to study the effect of supplemented carbon source on 2C4NP degradation. For these biodegradation experiments, strain CNP-8 was initially grown in MSM + lysogeny broth (LB) (4:1, *v/v*) containing 0.3 mM of 2C4NP. After overnight growth, cells were harvested, washed twice and resuspended in fresh MSM. The cells suspension was added in the test medium at initial OD_600_ of 0.05, and the flasks were then shaken at 180 rpm. All above experiments were performed in triplicate.

## 2C4NP degradation kinetics

To estimate the kinetics parameters of 2C4NP degradation, the effect of initial 2C4NP concentration (0.05–2 mM) on growth of strain CNP-8 was investigated. Cell growth kinetics was modeled by the following equation as described (Wang et al. 2010):

1 $$\mu = \frac{{\ln \left( {X/X_{0} } \right)}}{{t - t_{0} }}$$

where *X* represents the biomass (mg/L), *µ* represents the specific growth rate (h^−1^) and t represents the time.

Haldane’s model, widely used to describe the growth kinetics of toxic compounds (Shen et al. 2009; Banerjee and Ghoshal 2011), was selected to investigate the growth kinetics of 2C4NP. The Haldane’s inhibitory growth kinetics equation is as follows:

2 $$\mu = \frac{{\mu_{\text{max} } S}}{{K_{s} + S + \left( {S^{2} /K_{i} } \right)}}$$

where *S* represents substrate concentration (mg/L), *µ*_max_ represents the maximum specific growth rate (h^−1^), *K*_s_ represents half saturation constant (mg/L), *K*_i_ represents inhibition constant (mg/L).

The biomass yield coefficient (mg dry cell/mg 2C4NP) was calculated by the following equation:

3 $$Y = \frac{{X_{\text{max} } - X_{0} }}{{S_{0} - S_{a} }}$$

where *X*_max_ represents the maximum biomass concentration (mg/L); *X*_0_ represents initial biomass concentration; *S*_0_ represents initial substrate concentration (mg/L); *S*_a_ represents substrate concentration when biomass concentration reached maximum. The dry weight of biomass was converted from the OD_600_ value using a standard curve (dry weight (mg/L) = 564.7 × OD_600_ − 2.75, R^2^ = 0.986).

### Analytical methods

High performance liquid chromatography (HPLC) performed on an Agilent 1200 system was carried out to determine the concentration of 2C4NP and its metabolites with the mobile phase as described (Min et al. 2014). 2C4NP and the metabolites were quantified at 280 nm. For gas chromatography–mass spectrometry (GC–MS) analysis, the intermediates were acetylated as described (Perry and Zylstra 2007). The condition of GC–MS analysis was the same as described (Liu et al. 2010). The acetylated derivatives of the intermediates were identified based on comparisons of the mass spectra and GC retention times with those of the acetylated standards. Nitrite was assayed as described (Lessner et al. 2002). Chloride ion was detected with an ion-selective combination chloride electrode (Model 96-17, Orion, Boston).

### Biotransformation and enzymatic assay of the crude cell extract

Strain CNP-8 was grown in LB or MSM + LB (4:1, *v/v*) containing 0.3 mM 2C4NP, the cells were harvested at the exponential growth phase, washed twice and resuspended in 20 mM phosphate buffer (pH 7.2) to OD_600_ of 2.0. Biotransformation was performed with addition of 0.4 mM of substrates. The crude cell extract was prepared by ultrasonication followed by a centrifugation at 4 °C for 40 min at 20,000×*g*. The catalytic activity of the cell extract against 2C4NP, PNP and BT was determined spectrophotometrically. For 2C4NP and PNP transformation, the reaction mixture contained 20 mM phosphate buffer (pH 7.2), crude cell extracts, substrate (2C4NP or PNP, 20 μM), NADPH (100 μM), FAD (10 μM) and MgSO_4_ (1 mM) in a final volume of 500 μl. The reaction mixture for BT transformation contained 20 mM phosphate buffer, crude cell extracts and 100 μM of BT. In these enzymatic assays, the reference cuvette contained all components except the substrate, and activity assay was initiated with the addition of substrate.

### Chemotaxis of strain CNP-8

The chemotaxis of strain CNP-8 toward (chloro)nitrophenols was investigated as described (Arora and Bae 2014), with minor modification. For drop plate assay, strains SJ98 was grown in MSM + LB (*v/v *= 4:1) to exponential phase, and then induced by 0.3 mM of target substrate (2C4NP, 2C5NP, PNP or MNP) for 8 h. The cells were harvested by centrifugation at 4000×*g* for 10 min, washed twice with MSM, resuspended in the drop plate assay medium (MSM with 0.3% bacto agar) and transferred into petri-plate. Crystals of (chloro)nitrophenols were placed at the center of the plate and then incubated at 30 °C for 8–12 h. For quantitative capillary assay, the solutions of each substrate at different concentrations (from 0.05 to 0.7 mM in chemotaxis buffer which contained 100 mM potassium phosphate (pH 7.0) and 20 μM EDTA) were filled into 10 μl glass capillaries. The capillaries were inserted into the cell suspension of strain CNP-8 (~ 10^11^ cells/mL) on a glass slide. The capillary tube containing chemotaxis buffer [100 mM potassium phosphate (pH 7.0) with 20 μM EDTA] was used as control. After 30 min incubation at 30 °C, the solutions in the capillaries were diluted serially and spread onto LB plates. The total number of colony forming units (CFUs) was determined after 48 h incubation at 30 °C. The chemotaxis index (CFUs produced from capillary containing the target substrate/CFUs from a control capillary) was used to quantify the chemotactic response.

### Soil microcosm study

The soil was collected from an agricultural loam soil on the campus of the Yantai College of China Agricultural University, without pesticide contamination history. The total organic carbon, total organic nitrogen and moisture contents of the soil were 0.86, 0.074, and 10.64%, respectively, and the pH was 7.38. Soil in the sterile groups was sterilized three times by autoclaving at 121 °C for 30 min. Microcosms were set up in 250-mL glass bottles containing 110.6 g wet weight of soil [100 g dry weight (dw)]. Six different treatments were set up in triplicate: (T1) native soil with 2C4NP; (T2) sterilized soil with 2C4NP; (T3) native soil with 2C4NP and un-induced strain CNP-8; (T4) native soil with 2C4NP and 2C4NP-induced strain CNP-8; (T5) sterilized soil with 2C4NP and un-induced strain CNP-8; (T6) sterilized soil with 2C4NP and 2C4NP-induced strain CNP-8. 2C4NP with final concentrations of 100 μg/g dw was added to the soil and mixed thoroughly. For bioaugmentation, strain CNP-8 was grown in LB or MSM + LB (4:1, *v/v*) containing 0.3 mM of 2C4NP. Both the un-induced and induced cells were harvested at the exponential phase, cells were washed twice and resuspended in 0.85% sterile saline before being inoculating at ~ 1×10^9^ CFUs/g dw. An equivalent volume of sterile saline was added to microcosms not containing strain CNP-8. The bottles were thoroughly mixed and kept at 30 °C. Then, 0.5 g of soil samples was taken periodically. The method described previously (Niu et al. 2009) was followed to extract 2C4NP from the soil samples, and 2C4NP was quantified by HPLC analysis as described above.

## Results

### 2C4NP degradation and bacterial growth

**Fig. 1:**
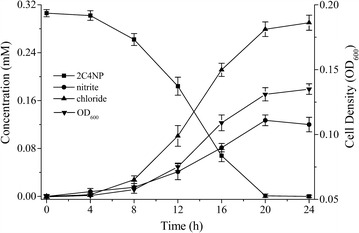
Degradation of 2C4NP by *Cupriavidus* sp. strain CNP-8, together with the accumulation of nitrite, chloride and the bacterial biomass (indicated by OD_600_)

Strain CNP-8, a member of the genus *Cupriavidus* (Genbank number of 16S rRNA sequence: KY643479) was isolated from pesticide-contaminated soil in Yantai, China. It has been deposited in the China Center for Type Culture Collection (Wuhan) with accession number CCTCC M 2017546. It completely degraded 0.3 mM of 2C4NP after 20 h incubation with release of nitrite and chloride ions. Meanwhile, the OD_600_ increased from initial 0.052 to final 0.135 (Fig. 1). This revealed that strain CNP-8 has the ability to utilize 2C4NP as sole source of carbon, nitrogen and energy, and the cell growth was closely correlated with the amount of 2C4NP utilized. Particularly, the amount of nitrite was found to be higher than that of chloride ion at each time-point when (NH_4_)_2_SO_4_ (1 g/L) was added into the MSM (data not shown), implying that the release of nitro group occurred prior to the removal of chloride. Moreover, the growth of strain CNP-8 on 2C4NP had a lag phase of about 4 h, indicating that strain CNP-8 had an induction period during 2C4NP degradation. In addition to 2C4NP, strain CNP-8 was also able to utilize 2C5NP and MNP; but it unable to degrade PNP, 4C2NP and 5C2NP (Additional file 1: Table S1).

### Effect of temperature, pH and additional glucose concentration on 2C4NP biodegradation

Biodegradation of 2C4NP by strain CNP-8 at different temperatures (20–40 °C) was examined when the pH of the MSM was fixed at 7. Strain CNP-8 had the highest efficiency of 2C4NP degradation at 30 °C, with rate of approximate 14.3 μM/h. The degradation rate declined by 32% when the culture temperature was increased to 35 °C, and declined by 25% when the temperature was reduced to 25 °C (Table 1). The 2C4NP degradation rate further reduced with the temperature increasing or decreasing; nevertheless, strain CNP-8 at a high temperature of 40 °C and a low temperature of 20 °C was still able to degrade 0.3 mM of 2C4NP completely within 62 and 57 h, respectively.

**Table 1:** Effect of temperature, pH, and glucose addition on 2C4NP degradation by strain CNP-8

Abiotic factors		OD_600_^d^	Time required for complete decolorization of 2C4NP (h)	Degradation rate (μM/h)^e^
Temperature (°C)^a^	20	0.105 ± 0.007	57 ± 3.1	5.3
	25	0.114 ± 0.008	28 ± 1.9	10.7
	30	0.137 ± 0.009	21 ± 2.2	14.3
	35	0.092 ± 0.006	31 ± 2.6	9.7
	40	0.073 ± 0.012	62 ± 4.5	4.8
pH^b^	4	ND	ND	ND
	5	0.112 ± 0.016	32 ± 2.6	9.4
	6	0.127 ± 0.014	22 ± 1.4	13.6
	7	0.135 ± 0.008	20 ± 2.5	15.0
	8	0.124 ± 0.011	23 ± 1.8	13.0
	9	0.103 ± 0.009	38 ± 2.7	7.9
	10	0.095 ± 0.014	54 ± 3.8	5.6
Glucose addition (g/L)^c^	–	0.133 ± 0.010	21 ± 1.8	14.3
	0.2	0.227 ± 0.014	12 ± 2.4	25.0
	0.5	0.342 ± 0.024	16 ± 1.6	18.8
	5	1.476 ± 0.038	49 ± 5.4	6.1

MSM containing 0.3 mM of 2C4NP was inoculated by strain CNP-8 with initial OD_600_ about 0.05 and shaken at 180 rpm

^a^pH of MSM was 7

^b^Culture temperature was 30 °C

^c^pH of MSM was 7 and culture temperature was 30 °C

^d^OD_600_ was determined at the time of complete decolorization of 2C4NP

^e^Rate of 2C4NP degradation (μM/h^−1^) = 300/time required for complete decolorization of 2C4NP

To investigate the effect of pH on 2C4NP degradation, strain CNP-8 was inoculated into MSM with pH ranging from 4 to 10. The cultural temperature was fixed at 30 °C. Strain CNP-8 exhibited the highest degradation rate against 2C4NP at pH 7. The degradation rates at pH 6 and 8 were slightly lower than that at pH 7. Although the degradation rates at pH 5, 9 and 10 dropped significantly, strain CNP-8 was still able to degrade 2C4NP completely (Table 1). Neither 2C4NP degradation nor cell growth was detected at pH 4. The special pH adaptability indicated that strain CNP-8 can be used under different conditions without adjusting the pH values.

Glucose with different concentrations (0.2, 0.5 or 5 g/L) was added to MSM with 0.3 mM of 2C4NP to investigate the effect of supplemental carbon source on 2C4NP degradation. Glucose with concentrations of 0.2 and 0.5 g/L can accelerate 2C4NP degradation, and the degradation rate of 0.2 g/L addition was higher than that of 0.5 g/L addition (Table 1). Although the biomass increased remarkably when the concentration of glucose was increased up to 5 g/L, the 2C4NP degradation rate declined significantly. This indicated that addition of easily degraded substrate is beneficial for 2C4NP biodegradation, but it is in a dose dependent manner.

### Kinetic studies of 2C4NP degradation by strain CNP-8

**Fig. 2:**
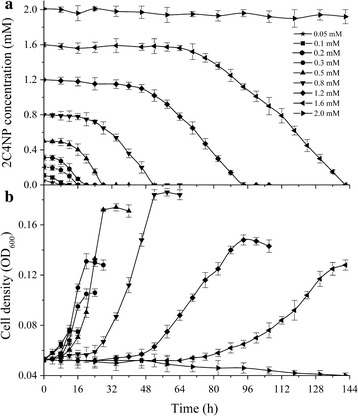
Biodegradation of 2C4NP (**a**) and growth of strain CNP-8 (**b**) at different 2C4NP concentrations. The experiments were performed in triplicate, and values are mean ± standard deviation (n = 3)

Degradation of 2C4NP at different concentrations (0.05–2 mM) was carried out to determine the kinetic parameters of 2C4NP biodegradation. As shown in Fig. 2, strain CNP-8 degraded 2C4NP completely when the substrate concentration was less than 1.6 mM. Both substrate degradation and cell growth were gradually delayed with increase of 2C4NP concentration. Neither substrate degradation nor cell growth was observed when the concentration of 2C4NP was increased up to 2 mM, suggesting that 2C4NP concentrations in excess of 2 mM would completely inhibit the growth of strain CNP-8.

**Fig. 3:**
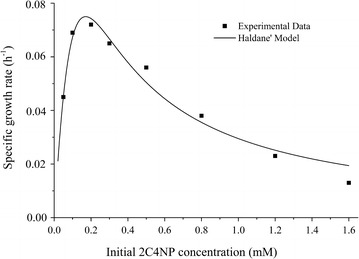
Experimental and predicted (Haldane’s model) specific growth rates of strain CNP-8

Haldane’s model, widely used to investigate microbial growth kinetics on aromatic compounds (Shen et al. 2009; Wang et al. 2010; Sahoo et al. 2011), was used to estimate the kinetic parameters of 2C4NP biodegradation. The values of kinetic parameters for 2C4NP degradation obtained here were *μ*_max_ = 0.148/h, *K*_s_ = 0.022 mM, *K*_i_ = 0.72 mM (R^2^ = 0.955, standard deviation = 0.067). The specific growth rate increased with increase of 2C4NP when the substrate concentrations were less than 0.16 mM (Fig. 3). At concentrations beyond 0.16 mM, a considerable decline in the specific growth rate was observed by increasing the 2C4NP concentration up to 1.6 mM. On the other hand, the biomass yield coefficient varied from 0.109 to 0.505 mg/mg when the initial 2C4NP concentration changed from 0.1 to 1.6 mM. The biomass yield coefficient changed slightly at low 2C4NP concentrations, and the highest yield coefficient (0.505 mg/mg) was obtained at a 2C4NP concentration of 0.3 mM. Further increases in 2C4NP concentration led to significant decrease in the values of yield coefficient, apparently due to the inhibitory effect of 2C4NP against strain CNP-8.

### Identification of the metabolites of 2C4NP degradation

The metabolites of 2C4NP degraded by strain CNP-8 were identified by HPLC and GC–MS analyses. No intermediate was detected when the sample was collected at 0 and 4 h. Metabolite I with HPLC retention time of 5.18 min was captured in the sample of 12 h (Additional file 1: Figure S1). It was identified as CHQ by comparison of the retention time with that of the standard. In the sample of 16 h, metabolite II with retention time of 8.07 min was also detected and it was identified as BT. Furthermore, GC–MS analysis of the acetylated products also detected two compounds, which were identified as acetylated CHQ (*m/z* 228: molecular ion peak; *m/z* 186: loss of one –COCH_3_; *m/z* 144: loss of two –COCH_3_) and acetylated BT (*m/z* 252: molecular ion peak; *m/z* 210: loss of one –COCH_3_; *m/z* 168: loss of two –COCH_3_; *m/z* 126: loss of three –COCH_3_), respectively (Additional file 1: Figure S2B and D). The identity of CHQ and BT was further confirmed by comparison with GC–MS analysis of the acetylated authentic compounds (Additional file 1: Figure S2A and C). On the basis of initial HPLC and GC–MS characterization, strain CNP-8 was proposed to degrade 2C4NP with BT as a ring-cleavage compound.

### Whole cell biotransformation and cell extract assays

Generally, the enzymes involved in the degradation of chlorinated nitroaromatic compounds were either inducible (Min et al. 2014, 2016) or constitutive (Gao et al. 2016). In this study, whole-cell biotransformation showed that the un-induced strain CNP-8 exhibited negligible activity for 2C4NP. However, the 2C4NP-induced cells degraded 2C4NP rapidly with a rate of approximately 0.271 mM/h OD_600_/cell, indicating that the enzymes involved in 2C4NP degradation in strain CNP-8 were inducible. Both CHQ and BT were detected during 2C4NP degradation. However, 2C4NP-induced cells of strain CNP-8 showed an evidently high rate of BT conversion (approximately 0.34 mM/h OD_600_/cell) than the cells converting CHQ (approximately 0.012 mM/h OD_600_/cell). Moreover, most previous 2C4NP-utilizers (Arora and Jain 2012; Min et al. 2014, 2016) were also able to degrade PNP and the catabolism of 2C4NP and PNP likely share the enzymes in these strains. However, the biotransformation assay showed that the 2C4NP-induced strain CNP-8 exhibited no activity for PNP (data not shown).

In the enzymatic assay, no activity of the cell extract against 2C4NP was observed when strain CNP-8 was grown in LB without 2C4NP induction. In contrast, the extract from the induced-cell transformed 2C4NP (λ_max_ = 403 nm) rapidly, together with the consumption of NADPH (λ_max_ = 340 nm) (Additional file 1: Figure S3A). Nitrite was also detected with the transformation of 2C4NP (data not shown). PNP transformation was not observed by the cell extract, in line with the result of the biotransformation. Degradation of BT by the cell extract also occurred, together with the formation of a new product with a maximum absorption at around 320 nm (Additional file 1: Figure S3B). This further proved that strain CNP-8 degraded 2C4NP via the BT pathway, apart from the identification of intermediate by HPLC and GC–MS assays.

### Chemotactic assays of strain CNP-8

**Fig. 4:**
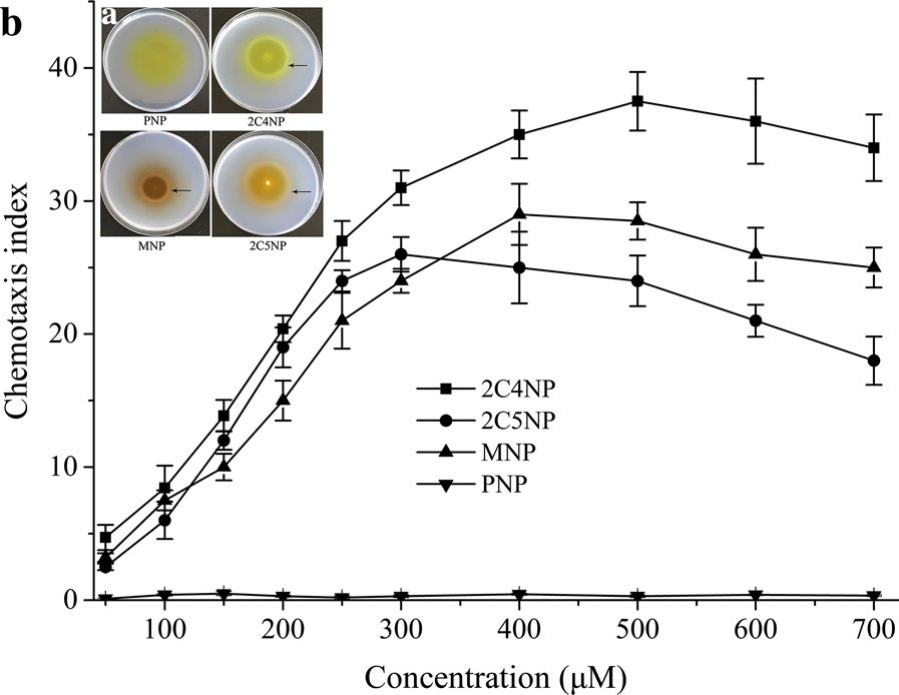
Drop plate (**a**) and capillary assays (**b**) of chemotaxis of strain CNP-8 towards different (chloro)nitrophenols. The arrows in **a** are meant to indicate the chemotaxis rings. In the capillary assays, the average number of CFUs produced from the control capillaries was 3.17 × 10^4^. The capillary assays were performed in triplicate, and values are mean ± standard deviation (n = 3)

We also investigated the chemotactic behavior of strain CNP-8 toward various (chloro)nitrophenols by both qualitative and quantitative assays. Drop plate assay showed that strain CNP-8 exhibited positive chemotaxis toward 2C4NP, 2C5NP and MNP, but showed no chemotactic response toward PNP (Fig. 4a). In the capillary assay, strain CNP-8 exhibited chemotaxis toward 2C4NP with a maximum chemotaxis index of 37.5 when the concentration of 2C4NP was 0.5 mM. The values of the chemotaxis index increased gradually with increase of 2C4NP up to the optimal concentration (Fig. 4b), and then remained relatively stable for further increase in substrate concentration. When the concentration of 2C5NP and MNP was 0.3 and 0.4 mM, respectively, strain CNP-8 exhibited the strongest chemotactic response toward these two chemoattractants, with chemotaxis index of 25.8 and 29.2, respectively. In contrast to these three compounds, strain CNP-8 exhibited no chemotactic response toward PNP at any concentration (Fig. 4b), in line with the result of drop plate assay.

### Bioremediation of 2C4NP-contaminated soil by strain CNP-8

**Fig. 5:**
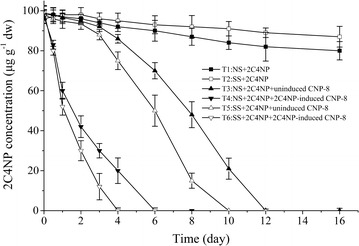
Biodegradation of 2C4NP by strain CNP-8 in soil microcosms. The experiments were performed in triplicate, and values are mean ± standard deviation (n = 3). *NS* native soil, *SS* sterilized soil

The capability of strain CNP-8 to remediate 2C4NP-contaminated soil was investigated by setting up 6 different microcosm using both nonsterile and sterile soils. Degradation of 2C4NP occurred rapidly in inoculated treatments (T3, T4, T5 and T6) than in the un-inoculated treatments (T1 and T2) (Fig. 5). In the inoculated non-sterile treatment T3 and inoculated sterile treatment T5, 2C4NP was completely removed on day 12 and 10, respectively. However, there was still 2C4NP in the un-inoculated non-sterile soil (T1) and un-inoculated sterile soil (T2) after 16 days of incubation, with 82% (80.3 ± 4.6 μg/g dw) and 91% (87.5 ± 5.2 μg/g dw) of the initial 2C4NP remained, respectively. It is obvious that the inoculated strain CNP-8 played crucial role in removing 2C4NP in the contaminated soil, whereas the indigenous microorganisms seemed to have no ability to degrade 2C4NP. The 2C4NP degradation rate in inoculated non-sterile soil (T3) is lower than that in inoculated sterile soil (T5). When un-induced cells of strain CNP-8 were inoculated in non-sterile soil (T3) and sterile soil (T5), the removal of 2C4NP has a lag phase of about 3 days (Fig. 5). However, degradation of 2C4NP in the inoculated induced groups (T4 and T6) had no delay period. So, it is our view that the pre-induced strain CNP-8 is more suitable for the bioremediation of 2C4NP-contaminated soil.

## Discussion

Several pure bacterial cultures have been isolated based on their ability to degrade 2C4NP (Ghosh et al. 2010; Arora and Jain 2011; Pandey et al. 2011; Arora and Jain 2012; Tiwari et al. 2017), but strain CNP-8 reported in present study is able to tolerate and degrade higher concentrations of 2C4NP than any other 2C4NP-utilizers. Our 2C4NP-degrading isolate can utilize 2C4NP up to concentration of 1.6 mM, while 2C4NP more than 0.5 mM has been reported to completely inhibit the growth of previously reported 2C4NP-utilizers including *Burkholderia* sp. RKJ800 (Arora and Jain 2012) and *Cupriavidus* strain a3 (Tiwari et al. 2017). Moreover, strain CNP-8 can adapt to a wide range of pH from 5 to 10, whereas strain a3 was reported to be unable to degrade 2C4NP at pH lower than 6 or higher than 9 (Tiwari et al. 2017). On the other hand, strain CNP-8 is also capable to utilize 2C5NP, which is the isomer of 2C4NP and usually coexist with 2C4NP in the industrial wastewater. However, there are no documented cases of 2C5NP degradation by other 2C4NP-degrading bacteria. From a practical point of view, these combined advantages of strain CNP-8 indicated that it could be a promising candidate for bioremediation of chloronitrophenols-contaminated environment.

The kinetic parameters of microbial pollutant degradation can enhance our understanding of the capability of degrading microorganisms and help us to compare the degradation efficiency among different isolates (Shen et al. 2009). The utilization of 2C4NP by strain CNP-8 followed the typical Haldane substrate inhibition model, in line with the reports of microbial degradation of numerous toxicants including phenol (Wang et al. 2010; Zhai et al. 2012; Basak et al. 2014), *p*-cresol (Singh et al. 2008), chlorobenzene (Zhang et al. 2011) and 4-chlorophenol (Sahoo et al. 2011). The value of biomass yield coefficient decreased at high 2C4NP concentration, likely due to that more energy was required to overcome the substrate inhibitory effect when the initial 2C4NP concentration was too high. Moreover, the accumulation of nitrite is likely another possible factor resulting in the decrease of the biomass yield coefficient when the concentration of 2C4NP more than 0.3 mM. Although there is a substrate inhibition, the evidently higher value of *K*_s_ (substrate affinity) than *K*_i_ (substrate inhibition) during 2C4NP degradation indicated that strain CNP-8 could efficiently degrade 2C4NP from the kinetic perspective. Moreover, strain CNP-8 had higher 2C4NP affinity and degradation efficiency than another 2C4NP-utilizer *Cupriavidus* strain a3 (Tiwari et al. 2017) by comparing the kinetic parameters of 2C4NP degradation.

Microbial chemotaxis was proposed to be helpful for bioremediation because previous studies have proved that chemotaxis can enhance the bioavailability of pollutants and/or promoted microbial consortia of various microorganisms with complementary degradation capabilities (Gordillo et al. 2007; Pandey et al. 2012; Arora and Bae 2014). In current study, it is clear that strain CNP-8 exhibited chemotaxis toward 2C4NP, 2C5NP and MNP, as evidenced by the formation of concentric chemotaxis rings and the determination of the chemotaxis indexes. Several 2C4NP-utilizers has been isolated (Ghosh et al. 2010; Arora and Jain 2011; Pandey et al. 2011; Arora and Jain 2012; Tiwari et al. 2017), but only *Burkholderia* sp. strain SJ98 exhibited chemotaxis toward 2C4NP, and its chemotaxis toward 2C5NP and MNP was not reported (Pandey et al. 2012). *Pseudomonas* sp. JHN showed chemotaxis toward 4C2NP (Arora and Bae 2014) and 4-chloro-3-nitrophenol (Arora et al. 2014b), two isomers of 2C4NP; however, it showed no chemotactic behavior toward 2C4NP and 2C5NP. To date, both metabolism-dependent chemotaxis and metabolism-independent chemotaxis of aromatic compounds have been studied in bacteria (Zhang et al. 2008; Arora and Bae 2014). The chemotaxis of strain CNP-8 towards (chloro)nitrophenols is likely a typical metabolism-dependent chemotaxis, as evidenced by the fact that strain CNP-8 only exhibited chemotaxis toward the compounds which it can utilize.

**Fig. 6:**
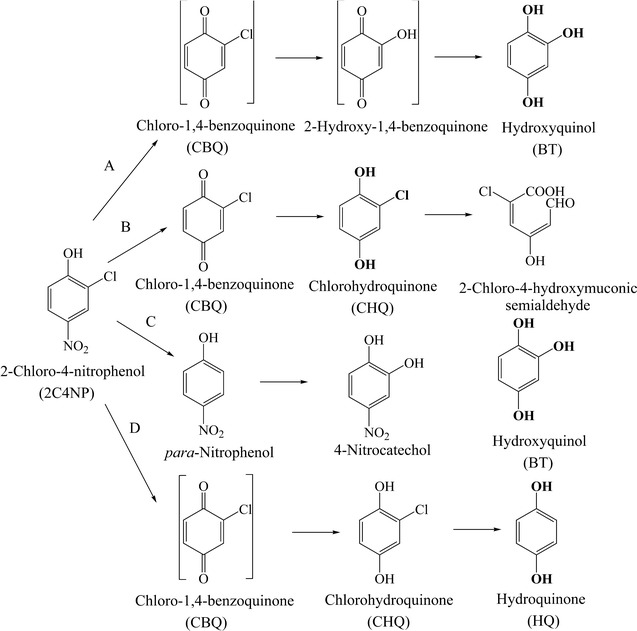
Proposed pathways of 2C4NP degradation by 2C4NP utilizers. (A) *Cupriavidus* sp. strain CNP-8 (this pathway was also seen with *Rhodococcus imtechensis* strain RKJ300, reported previously); (B) *Burkholderia* sp. strain SJ98 [reported by Min et al. (2014)]; (C) *Burkholderia* sp. strain SJ98 [reported by Pandey et al. (2011)]; (D) *Burkholderia* sp. strain RKJ800. The unidentified intermediates are in brackets. The structures of ring cleavage compounds are indicated in boldface

The detection of CHQ indicated that the degradation of 2C4NP by strain CNP-8 was initiated by a 2C4NP 4-monooxygenase. It is well known that a monooxygenase attack on an aromatic ring at a position substituted by an electron-withdrawing group forms a quinone compound (Entsch et al. 1980; Haigler et al. 1996; Xun and Webster 2004; Perry and Zylstra 2007), while an attack at an unoccupied position forms a quinol compound (Whited and Gibson 1991; Tao et al. 2004). Therefore, the first intermediate of catabolism of 2C4NP was proposed as chloro-1,4-benzoquinone (CBQ). The identification of BT suggested that CBQ was subsequently dechlorinated to BT likely via 2-hydroxy-1,4-benzoquinone (Fig. 6A), similar with the catabolism of 2,4,6-trichlorophenol in *Cupriavidus necator* JMP134 (Xun and Webster 2004). Both CBQ and 2-hydroxy-1,4-benzoquinone were not detected during 2C4NP degradation is likely due to that they were easily reduced to CHQ and BT, respectively by nonenzymatic transformation, in line with the hypotheses of nonenzymatic reduction of other quinone compounds (Xun and Webster 2004; Perry and Zylstra 2007; Zhang et al. 2009; Yamamoto et al. 2011; Min et al. 2014; 2016). This speculation was further justified by the detection of CHQ during 2C4NP degradation. CHQ may not be a true intermediate because the 2C4NP-induced cells of strain CNP-8 hardly transformed CHQ.

The 2C4NP catabolic pathway identified in strain CNP-8 is different from those reported in other 2C4NP utilizers. Pandey et al. have reported that *Burkholderia* sp. strain SJ98 initiated 2C4NP degradation with formation of *para*-nitrophenol, which was then transformed to BT via 4-nitrocatechol (Fig. 6C) (Pandey et al. 2011); however, subsequent genetic and biochemical identification suggested CHQ pathway is likely the actual pathway of 2C4NP catabolism in this strain (Fig. 6B) (Min et al. 2014). In this study, BT was identified as the ring cleavage substrate during 2C4NP degradation by strain CNP-8. This clearly reveals that the removal of chloro group occurs before ring cleavage during 2C4NP catabolism in strain CNP-8, whereas the chloro group was removed after ring cleavage in strain SJ98. Although the removal of chloro group also occurs before ring cleavage during 2C4NP degradation by *Burkholderia* sp. RKJ800 (Fig. 6D), HQ rather than BT was identified as the ring cleavage substrate (Arora and Jain 2012). *Rhodococcus imtechensis* RKJ 300, a Gram-positive 2C4NP-utilizer, was reported to degrade 2C4NP with BT as a ring cleavage substrate, which was then transformed to maleylacetate (λ_max_ = 243 nm) by BT dioxygenase (Min et al. 2016). In present study, although the ring fission product of BT was not identified, enzymatic assay clearly showed that the product of BT had a λ_max_ of 320 nm (Additional file 1: Figure S3B), apparently different from the spectral property of maleylacetate. To our knowledge, this is the first report of 2C4NP degradation via the BT pathway in a Gram-negative 2C4NP utilizer, and this finding could enhance our understanding of the diversity of metabolic pathways of microbial 2C4NP degradation.

More interestingly, most 2C4NP-utilizers including *Burkholderia* sp. strain SJ98 (Min et al. 2014), *Burkholderia* sp. strain RKJ800 (Arora and Jain 2012) and *Rhodococcus imtechensis* RKJ 300 (Ghosh et al. 2010) were also able to grow on PNP. In particular, both the single-component 2C4NP monooxygenase (PnpA) from the Gram-negative strain SJ98 (Min et al. 2014) and the two-component 2C4NP monooxygenase (PnpA1A2) from the Gram-positive strain RKJ 300 (Min et al. 2016) were reported to be able to catalyze the monooxygenation of 2C4NP and PNP. In contrast, enzymatic assay in present study clearly revealed that the monooxygenase involved in 2C4NP catabolism in strain CNP-8 was unable to catalyze the transformation of PNP, indicating its special substrate spectrum as compared to other reported 2C4NP monooxygenations. Now, the purification and characterization of enzymes involved in 2C4NP catabolism in strain CNP-8 is in progress, and the genetic characterization of strain CNP-8 will be the subject of further work.

## Additional file

**Additional file 1: Table S1.** Degradation capability of strain CNP-8 for various nitrophenols. **Figure S1.** HPLC identification of the intermediates of 2C4NP degradation by strain CNP-8. **Figure S2.** Mass spectra of the acetylated derivatives of the intermediates during 2C4NP degradation by strain CNP-8. (A) Acetylated derivative of authentic CHQ. (B) Acetylated metabolite I. (C) Acetylated derivative of authentic BT. (D) Acetylated metabolite II. **Figure S3.** Transformation of 2C4NP (A) and BT (B) by the cell extract of 2C4NP-induced strain CNP-8.

## Abbreviations

2C4NP: 2-chloro-4-nitrophenol
2C5NP: 2-chloro-5-nitrophenol
BT: 1,2,4-benzenetriol (hydroxyquinol)
CHQ: chlorohydroquinone
CNPs: chloronitrophenols
HQ: hydroquinone
*K*_i_: substrate inhibition constant
*K*_s_: half saturation constant
PNP: *para*-nitrophenol
*μ*_max_: maximum specific growth rate
