# Supplementary material for: Biodegradation of 2-chloro-4-nitrophenol via a hydroxyquinol pathway by a Gram-negative bacterium, Cupriavidus sp. strain CNP-8
Source: AMB Express. 2018 Mar 20;8:43. doi: 10.1186/s13568-018-0574-7 (PMC5861257; doi:10.1186/s13568-018-0574-7)
Supplement: Supplementary file 1 — Additional file 1: Table S1. Degradation capability of strain CNP-8 for various nitrophenols. Figure S1. HPLC identification of the intermediates of 2C4NP degradation by strain CNP-8. Figure S2. Mass spectra of the acetylated derivatives of the intermediates during 2C4NP degradation by strain CNP-8. (A) Acetylated derivative of authentic CHQ. (B) Acetylated metabolite I. (C) Acetylated derivative of authentic BT. (D) Acetylated metabolite II. Figure S3. Transformation of 2C4NP (A) and BT (B) by the cell extract of 2C4NP-induced strain CNP-8. [file 13568_2018_574_MOESM1_ESM.pdf]

AMB Express

Biodegradation of 2-chloro-4-nitrophenol via a hydroxyquinol pathway by a gram-negative bacterium, *Cupriavidus* sp. strain CNP-8

Jun Min[1], Jinpei Wang[2], Weiwei Chen[1] and Xiaoke Hu[1*]

[1] Key Laboratory of Coastal Biology and Bioresource Utilization, Yantai Institute of Coastal Zone Research, Chinese Academy of Sciences, Yantai 264003, China

[2] Key Laboratory of Agricultural and Environmental Microbiology, Wuhan Institute of Virology, Chinese Academy of Sciences, Wuhan 430071, China

[*]Corresponding author: Xiaoke Hu; Email: xkhu@yic.ac.cn; Tel: 86-535-2109127; Fax: 86-535-2109127

Table S1 Degradation capability of strain CNP-8 for various nitrophenols.

Nitrophenols	Utilization of nitrophenols as carbon and nitrogen sources by strain CNP-8	
	Decolourization of MSM agar plate containing 0.3 mM of nitrophenol(s)	Growth on liquid MSM containing 0.3 mM of nitrophenol(s)
2C4NP	+	+
2C5NP	+	+
4C2NP	−	−
5C2NP	−	−
MNP	+	+
PNP	−	−

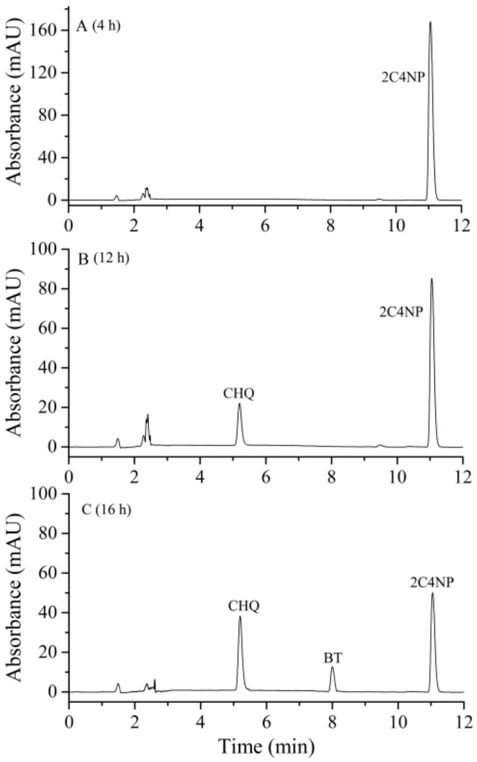

Fig. S1 HPLC identification of the intermediates of 2C4NP degradation by strain CNP-8.

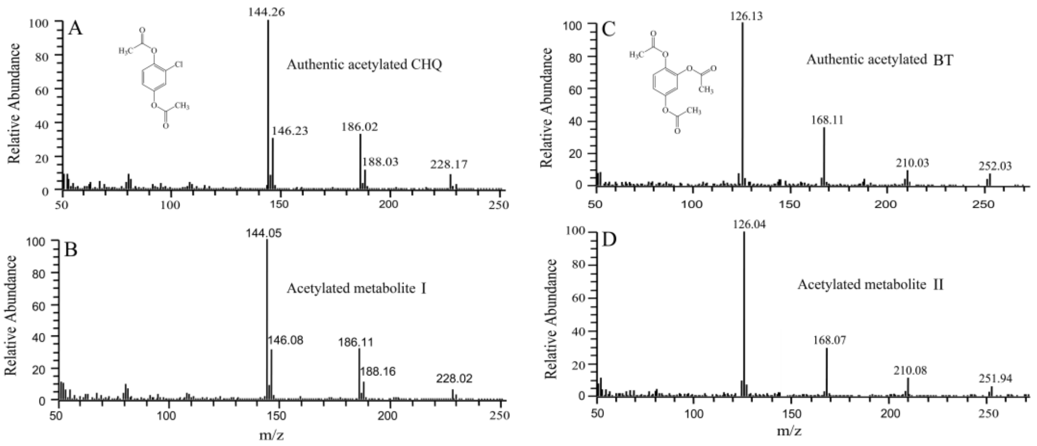

Fig. S2 Mass spectra of the acetylated derivatives of the intermediates during 2C4NP degradation by strain CNP-8. (A) Acetylated derivative of authentic CHQ. (B) Acetylated metabolite I. (C) Acetylated derivative of authentic BT. (D) Acetylated metabolite II.

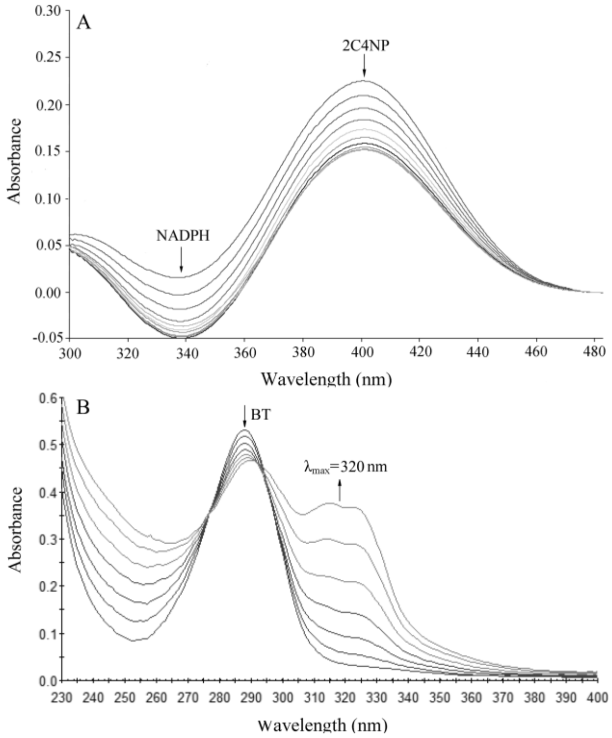

Fig. S3 Transformation of 2C4NP (A) and BT (B) by the cell extract of 2C4NP-induced strain CNP-8.
